# Finding the Sweet Spot for the Treatment of B Cell Malignancies

**DOI:** 10.3390/cancers17203366

**Published:** 2025-10-18

**Authors:** Valerie R. Wiersma

**Affiliations:** Department of Hematology, University Medical Center Groningen, University of Groningen, 9713 GZ Groningen, The Netherlands; v.wiersma@umcg.nl

**Keywords:** glycosylation, glycans, B cell leukemia, B cell lymphoma, lectins, immunotherapy

## Abstract

The glycan profile of cells comprises a high variety of sugar moieties that are attached to proteins (glycoproteins) and lipids (glycolipids) via a process called ‘glycosylation’. Cancer cells commonly carry aberrant glycans, which may be of interest for cancer diagnosis, prognosis, as well as the development of novel therapeutic strategies. This review focuses on the differential glycosylation patterns on malignant B cells, including both B cell lymphoma and leukemia. Well-known aberrant glycan profiles on malignant B cells include acquired high mannose N-glycans in the B cell receptor (BCR) of follicular lymphoma (FL), and increased expression of the glycosphingolipid Gb3/CD77 on Burkitt’s lymphoma (BL). These structures can be exploited for therapy by using lectins that specifically recognize these patterns with intrinsic cytotoxic activity or in a drug-conjugate format. Furthermore, immunotherapy can be improved by modulating glycans, especially sialic acids. Targeting glycans for immunotherapy is also of interest for chimeric antigen receptor (CAR) T cell therapy, a relatively novel therapy that has been quite effective in various B cell malignancies. Thus, the glycan profile of malignant B cells harbors many opportunities for therapeutic targeting. It is anticipated that further in-depth glycan profiling will open up many more opportunities for the treatment of B cell malignancies.

## Simple Summary

Most proteins and lipids in our cells are ‘decorated with sugars’. These sugar decorations commonly differ between cancer cells and healthy cells and are therefore of interest for therapeutic targeting. This review is focusing on sugar decorations that are present on B cell malignancies, including both B cell leukemia and lymphoma. Furthermore, potential ways to target these sugar decorations for the treatment of B cell malignancies are discussed. The final section describes the hurdles that still need to be taken before therapies that focus on the sugar decorations of B cell malignancies can be translated to the clinic.

## 1. Introduction

**Figure 1:**
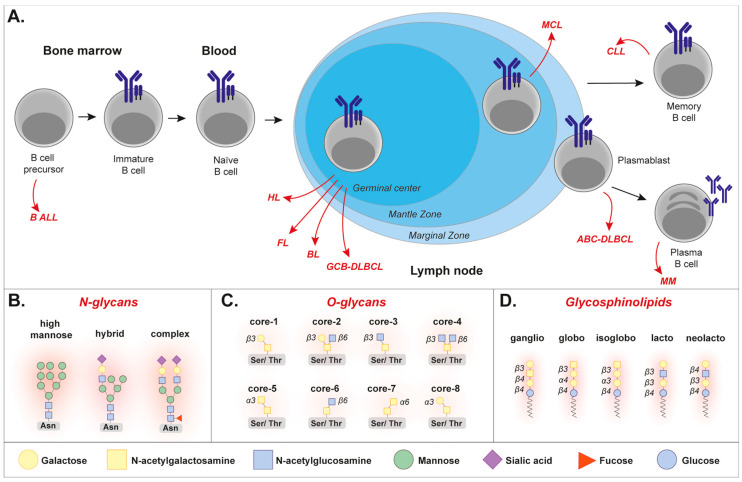
Overview of B cell malignancies and common types of glycosylation. (**A**) Various types of B cell malignancies exist, which originate from B cells at various maturation/differentiation statuses. The figure depicts the B-cell malignancies that are most frequently mentioned in this review: acute lymphoblastic leukemia (ALL), Hodgkin’s lymphoma (HL), follicular lymphoma (FL), Burkitt’s lymphoma (BL), mantle cell lymphoma (MCL), chronic lymphocytic leukemia (CLL), diffuse large B-cell lymphoma (DLBCL), germinal center B cell-like (GCB), and activated B cell-like (ABC). (**B**) The three major types of N-glycans. (**C**) The eight core structures of O-glycans. (**D**). The five major types of glycosphingolipids. Of note, throughout the whole manuscript, glycans will be depicted with a ‘red glow’ to emphasize them.

B cell malignancies comprise a wide range of cancers that originate from abnormal B lymphocytes. These cancers can be subdivided into lymphomas and leukemias based on their origin, being the lymphatic system for lymphoma and the bone marrow for leukemia. In addition, multiple myeloma is a B cell malignancy that arises in B cell plasma cells, and also originates from the bone marrow (Figure 1A). Although these malignancies are all B cells, their appearance and expression profile of protein markers are quite different between diseases [1,2,3], as well as compared to healthy B cells [4,5,6,7]. In addition to protein expression, the ‘sugar decoration’ of malignant B cells is of interest to study, especially in the context of developing novel therapeutic strategies.

The process of ‘glycosylation’, which takes place in the ER and Golgi (as reviewed by [8]), is a post-translational modification that gives rise to a high variety of glycoproteins and glycolipids. In humans, two main types of protein glycosylation exist, being N-glycosylation and O-glycosylation (as fully described in [9]). In brief, N-glycosylation is a process in which glycans are attached to the nitrogen atom of an asparagine residue (Asn), which only occurs at the consensus sequence Asn-X-Ser/Thr, whereby ‘X’ can be any amino acid except proline [9]. The initial sugar of N-glycans is always an N-acetylglucosamine (GlcNAc), which is further extended to form three major N-glycan types: high mannose, complex, and hybrid N-glycans (Figure 1B). In the case of O-glycosylation, the glycans are attached to the oxygen atom of a serine or threonine residue (Ser/Thr) [9]. O-glycans exist in eight different core structures, which all contain an initial N-acetylgalactosamine (GalNAc) attached to Ser/Thr (Figure 1C). A well-known type of glycolipid is the glycosphingolipid, which consists of a hydrophobic ceramide and a carbohydrate (Figure 1D). Glycosphingolipids are highly abundant in the plasma membrane of cells, where the lipids are inserted in the lipid bilayer and the attached glycans stick outside and participate in, for instance, cell–cell interactions. The addition of glycans to forming glycoproteins and glycolipids is performed by enzymes called glycosyltransferases. Their counterparts are glycosidases, which cleave off sugar moieties from glycan structures. Notably, the process of glycosylation is non-template driven, and is dynamically controlled by, among others, the glycosyltransferase/glycosidase levels, availability of precursor sugars, and the metabolic flux of the cell.

Glycans have important functions in B cells, for instance during B cell development, B cell differentiation, and regulating B cell survival [10,11,12]. Therefore, the glycans expressed by B cell malignancies may be interesting targets for cancer therapy. Hence, this review aims to find ‘the sweet spot’ for the treatment of B cell malignancies through: (1) focusing on differential and/or aberrant glycan profiles in B cell malignancies, (2) discussing how these different glycan profiles may be utilized for glycosylation-targeted therapy, and (3) elaborating on how to translate this glycan targeting to the clinic.

## 2. Aberrant Glycan Profiles in B Cell Malignancies

Aberrant glycosylation is a common feature of cancer cells; hence, several glycosylation-related biomarkers have already found their way to clinical practice [13]. Also, in B cell malignancies, various changes in glycosylation patterns have been detected, which commonly contribute to their survival, and therefore also correlate with patient outcome. In this section, the best-known deviations in glycosylation patterns in B cell malignancies will be described, focusing on (1) the acquired high mannose N-glycans in the B cell receptor (BCR) of malignant B cells, (2) the elevated levels of the glycosphingolipid globotriasosylceramide (Gb3/CD77) in Burkitt’s lymphoma (BL), and (3) hypersialylation in B cell lymphoma.

### 2.1. BCR-Stimulation via High Mannose N-Glycans in B Cell Lymphoma

#### 2.1.1. Acquired Highly Mannosylated N-Glycan Motifs in the BCR of Lymphoma Cells

The BCR is a transmembrane protein complex on the plasma membrane of all B cells that consists of an immunoglobulin (Ig) and CD79 (CD79a/b). Igs are heavily glycosylated proteins, whereby IgGs contain one conserved N-glycosylation site (Asn-297), whereas IgMs carry up to five potential N-glycosylation sites [14]. Continuous stimulation of the BCR is essential for the survival of B cell lymphoma cells, which is commonly maintained by engagement with self-antigens derived from the *IGHV4-34* gene [15,16,17]. At early stages of FL, somatic hypermutations and gene rearrangements occur, commonly resulting in the acquisition of novel N-glycosylation motifs, i.e., Asn-X-Ser/Thr, in the BCR. Specifically, multiple studies detected at least one acquired N-glycosylation motif in the BCR-heavy chain of FL samples, reaching a 100% detection rate in half of the studies (Table 1A). This is in contrast to the BCR-light chain, where an acquired N-glycosylation motif could be detected in only 0–59% of the FL samples (Table 1B). Most acquired N-glycosylation motifs in the Ig heavy chain were detected in complementarity-determining region 2 (CDR2) and CDR3, which corresponds to the fact that most hypermutation and recombination take place in these regions [18]. One study suggested that the location of Asn-X-Ser/Thr motifs is associated with BCR isotype. Specifically, the acquired N-glycosylation motifs in IgM molecules predominantly resided in the CDR2 region, whereas IgG-containing BCRs predominantly contained this motif within the CDR3 [19]. However, in another study, where 14 FL samples with an IgM-BCR were analyzed, eight samples contained at least one acquired N-glycosylation motif in the CDR3 region versus seven samples with these motifs in the CDR2 [20]. Hence, there is currently no clear evidence for isotype-specific regions of acquired N-glycosylation motifs. The acquired N-glycosylation motifs were already present in patient samples at the time of diagnosis, and were conserved in almost all subclone populations over time (>96%) [21]. Similarly, only 2 out of 17 patient samples lost their acquired N-glycosylation motifs over time in another study [20]. These findings correspond to the overall thought that these additional N-glycosylation sites are acquired at the onset of the disease. Even with ongoing somatic hypermutations, that further changed the amino acid composition in certain subclones, most sequences still encoded for an N-glycosylation motif. Interestingly, certain FL samples contained up to eight sequences that only required one additional mutation to become an acquired N-glycosylation motif [22]. Thus, the acquisition of novel N-glycosylation sites is a common phenomenon in FL and seems to contribute to tumor progression, as these mutations emerge at early onset, and are retained throughout all stages of disease in the vast majority of analyzed samples.

Acquired N-glycosylation motifs have also been detected in other lymphoma types, but commonly at lower levels than in FL. In this respect it should be noticed that also healthy B cells contain N-glycosylation motifs, which could be detected in 7 out of 75 samples (9%) [23]. Therefore, in order to differ from normal N-glycosylation motifs, the level of these motifs in malignant B cells should be above and differ significantly from 9%. After FL, BL is the lymphoma type with the highest amount of acquired N-glycosylation motifs (Table 1C), with samples containing at least one motif ranging from 25 to 82%. Thereafter, DLBCL is the lymphoma type with the highest amount of acquired N-glycosylation motifs, whereby 0–41% of the samples contained at least one motif (Table 1C). Here it should be noticed that the extent of acquired N-glycosylation motifs differs between subtypes, with 23 out of 180 (13%) in ABC-DLBCL, and 55 out of 92 (60%) in GCB-DLBCL [32]. Furthermore, the sample size is of importance, as studies that did not find any acquired N-glycosylation motif in DLBCL samples all had a very low sample size (Table 1C). The study with a large sample size found acquired N-glycosylation motifs in IgA, IgM, and IgG BCRs, predominantly in their CDR, CDR3, and FR3 regions, respectively, [32]. In addition, acquired N-glycosylation motifs were detected in other lymphoma types and MM, whereby the amount of motifs did not differ from healthy B cells in most disease subtypes. Interestingly, in the case of N-glycosylation-negative B cell lymphoma cells, these cells commonly rely on the *IGHV4-34* gene for their survival. Specifically, the *IGHV4-34* gene was more commonly found in ‘N-glycosylation negative’ FL [20] and ABC-DLBCL [32]. Also, t(14;18)-negative FL contained fewer acquired N-glycosylation motifs compared to t(14;18)-positive FL [33] at disease stage II/IV, and relied more on *IGHV4-34* for their survival [34]. Thus, acquired N-glycosylation motifs are commonly present in lymphoma subtypes, and especially FL.

**Figure 2:**
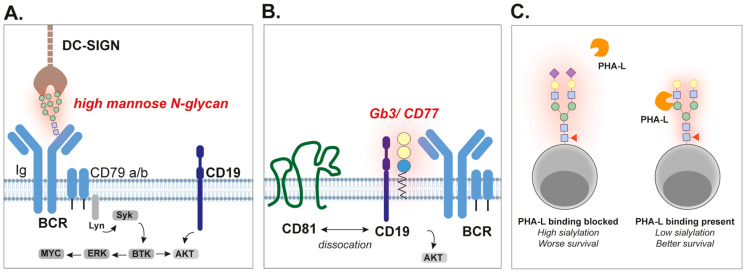
Aberrant glycans expressed on B cell malignancies. (**A**) Malignant B cells, and especially follicular lymphoma B cells, commonly have acquired N-glycans in their BCR. These N-glycans are highly mannosylated and provide BCR-stimulation via interaction with mannose-binding lectins like DC-SIGN. (**B**) Burkitt’s lymphoma B cells express very high levels (up to 100 times more than normal) of the glycosphingolipid globotriasosylceramide, also known as Gb3 or CD77, on their membrane. Gb3 dissociates CD81 from CD19, enabling CD19-mediated BCR signaling. (**C**) PHA-L is a lectin that binds to complex N-glycans, which is inhibited by α2,6-coupled sialic acids. DLBCL and BL patients with low PHA-L reactivity (and thus high sialylated PHA-L epitope) towards their biopsies had a worse overall survival.

Further characterization of the Ig-domain of the BCR of FL patients revealed that most of the N-glycans were highly mannosylated [25]. Specifically, oligomannose peaks were observed using NP-HPLC, corresponding to glycans carrying 5 to 9 mannose residues (M5–M9). Since the Fc region of the Ig samples did contain complex glycans, it was concluded that normal glycosylation pathways were intact, but that the variable regions of the FL Ig specifically carried these high-mannose glycans. Also, when comparing Igs from a selected FL patient to Igs from a healthy donor, an increase in the total amount of oligomannoses was observed, respectively, ~31% versus ~49%. Correspondingly, α-mannosidase digestion, which cleaves off all mannose residues, resulted in NP-HPLC profiles that looked very similar to the profile of Ig isolated from a CLL patient that lacked the acquired N-glycosylation motifs. Additional glycan analysis upon papain digestion, which separates the Fab-fragments from the Fc-domain, demonstrated that the additional mannoses were present in the variable region of the Ig. Treatment with Endo H, which cleaves asparagine-linked mannose-rich oligosaccharides from glycoproteins, induced a shift in Western blot protein migration of Ig in six out of seven (86%) FL samples [26] and GCB-DLBCL samples [32]. High mannose N-glycans were also detected in the BCR-IgM of CLL cells, although predominantly in the heavy chain constant region instead of the variable region [35]. Here, a high variability in the ratio between mature and immature highly mannosylated glycans was detected among the CLL samples, whereby CLL without a mutation in the variable domain of the Ig heavy chain harbored a higher amount of immature high mannosylated N-glycans. Interestingly, persistent stimulation of the BCR resulted in the loss of the mature glycoform form without affecting expression of the immature highly mannosylated form. Also, on normal B cells, which commonly do not express high levels of highly mannosylated Igs [36], the expression of a highly mannosylated glycoform could be induced upon persistent activation of their BCR [35]. Taken together, B cell lymphoma cells, and especially FL cells, commonly harbor acquired mannosylated N-glycosylation motifs in their BCR (Figure 2A).

#### 2.1.2. Highly Mannosylated N-Glycans Activate BCR-Signaling by Interacting with DC-SIGN

The high prevalence of highly mannosylated N-glycans in the BCR of B cell malignancies suggests that this motif provides pro-tumorigenic and/or survival signaling. Indeed, FL cells that are positive for acquired N-glycosylation motifs in their BCR had a higher activity of BCR and inflammatory signaling as compared to ‘N-glycosylation negative’ FL [20]. Additionally, increasing the level of mannosylation using JDW-010, a kifunensine derivative, slightly increased BCR signaling in B cells [37]. However, the introduction of N-glycosylation motifs into a TKO B cell line model strongly reduced the interaction of the BCR with its cognate antigen, thus impairing normal antigen stimulation [38]. Therefore, B cell malignancies with acquired N-glycan motifs seem to obtain BCR-stimulating signaling in an antigen-independent manner.

Interestingly, high mannose-binding lectins are highly present in the microenvironment of lymphoma cells, which may interact with the highly mannosylated BCRs. Indeed, mannose-binding lectin (MBL), secreted by cells of the innate immune system, bound stronger to samples that contained an acquired N-glycosylation motif in their CDR2 compared to a control that did not [25]. Also, innate immune cells express lectin-binding receptors, among which are the mannose receptor (MR, CD206) and dendritic cell-specific intercellular adhesion molecule-3-grabbing non-integrin (DC-SIGN, CD209). Of note, DC-SIGN could be detected in lymph nodes of FL patients, both within sinusoid-like structures as well as on mononuclear cells [27,39]. MR-lectin and DC-SIGN bound to both FL cells and normal B cells in vitro but only stimulated intracellular signaling in FL cells [36]. Furthermore, pre-incubation with these lectins prevented the binding of an anti-IgM antibody to FL cells but not healthy B cells, suggesting that the lectins interacted with the mannose residues present on the IgM molecule. In line with this data, DC-SIGN binding correlated with the level of surface IgM expression in FL samples [39]. Further characterization of the intracellular pathways in FL cells demonstrated that the interaction of DC-SIGN with IgM/IgG activated ERK1/2 and AKT [39] (Figure 2A). Correspondingly, normal B cells that lacked an acquired N-glycosylation motif did not activate the ERK/AKT pathway upon DC-SIGN stimulation, whereas anti-Ig did. Also, the downstream kinase SYK was phosphorylated in both FL and DLBCL upon DC-SIGN interaction [32,39], which could be prevented by both SYK inhibitors and a DC-SIGN blocking antibody [32].

Stimulation with either DC-SIGN or anti-Ig induced comparable gene expression signatures (~41–53% overlap in genes), although the effect induced by DC-SIGN was weaker as compared to anti-Ig [32,40]. Pre-incubation with DC-SIGN blocked ~80% of the effect induced by anti-Ig, again proving that both molecules act via the same BCR. Many genes related to BCR activation were upregulated, but also the oncogene MYC. This is in line with the increased expression of MYC in FL cells, but not normal B cells upon stimulation with DC-SIGN [39]. In contrast, DC-SIGN could not induce BCR activation, as determined by calcium flux measurements, in the TKO B cell model [38]. However, it should be noted that the buffer that was used in these experiments impacted the morphology of cells. Additionally, differences between the studies may originate from using primary FL cells versus in vitro cell models. In this respect, DC-SIGN was also unable to induce calcium flux in the WSU-FSCCL cell line (DLBCL origin) that does harbor an acquired N-glycosylation motif, even though it did activate ERK and AKT [40]. In contrast, mannose-binding lectins isolated from opportunistic bacteria did stimulate calcium fluxes in the TKO B cell line model [38]. Although these different responses in TKO cells may reflect limitations of the model cell line, it may also implicate that the strength of the stimulation of the BCR dictates its activation, as lectins are able to form lattices with strong overall avidity [41]. Indeed, DC-SIGN-Fc induced calcium flux in FL samples, whereas DC-SIGN-HA did not [40], which may stem from the ability of the Fc-domain to induce additional antibody-mediated cross-linking. Correspondingly, the impact of immobilized DC-SIGN was stronger than for soluble DC-SIGN [39]. Together this data suggests that in vitro analysis should be taken with caution, and that within the tumor microenvironment, where DC-SIGN is immobilized on the cell membrane of innate immune cells, the interaction between the BCR and DC-SIGN is likely strong enough to induce stimulatory signaling.

Whereas stimulation with anti-Ig induced endocytosis of surface-expressed Ig, stimulation with DC-SIGN did not [39]. BCR endocytosis is a well-known phenomenon upon BCR activation, although the exact function of this process is still unclear. On the one hand, BCR endocytosis seems to be required for effective downstream signaling, including appropriate kinase activation [42]. On the other hand, BCR endocytosis downregulates BCR signaling and increases the threshold for subsequent BCR activation [43]. Hence, it is tempting to speculate that this lack of endocytosis of the BCR upon ligation by mannose-binding lectins facilitates continued B cell stimulation. Indeed, the response induced by DC-SIGN, although weaker, persisted longer as compared to anti-Ig [39]. This weaker activation by DC-SIGN may also contribute to sustained BCR activation, as it prevents BCR overstimulation. In addition, samples from DLBCL patients with an acquired N-glycosylation motif in their CDR displayed an increase in gene expression related to BCR signaling as compared to samples without [32]. Thus, FL cells likely benefit from the acquired N-glycosylation motifs in their BCR by interacting with mannose-binding lectins that are present in their microenvironment. In this respect, DC-SIGN is highly expressed on tumor-supportive M2 macrophages [44], which may provide stimulatory signaling. In line with this hypothesis, FL patients with a high level of lymphoma-associated macrophages have a worse overall survival [45]. Interestingly, DC-SIGN expression on monocytes and macrophages is induced by IL-4 [46,47], a cytokine that is present at high levels in FL and well-known for its tumor-promoting effects [48,49,50]. Indeed, treatment with IL-4 induced DC-SIGN in monocyte-derived dendritic cells (moDCs), which formed clusters with DLBCL cells with highly mannosylated Ig, and not with samples that did not acquire this glycosylation modification [32]. Hence, IL-4 may provide tumorigenic signaling both directly as well as indirectly via the regulation of DC-SIGN expression.

The acquisition of N-glycosylation motifs is also associated with a ‘dark zone signature’ in FL cells. In normal B cell biology, B cells that reside in the germinal center (GC) undergo extensive hypermutation and proliferation in the dark zone, after which they move to the light zone. Here the cells that are positively selected either become plasma cells or memory B cells or return to the dark zone to undergo further mutations. When comparing gene expression profiles of FL cells from the same patient with or without an acquired N-glycosylation motif within their BCR, differences in expression profiles were predominantly found in the genes that distinguish naïve cells from dark zone cells [51]. Focused analysis within a smaller set of light- and dark zone-related genes, identified that 86% of the dark zone-related genes were significantly higher expressed in FL cells with an acquired N-glycosylation motif. On the contrary, 78% of the light zone-related genes were expressed at lower levels in these cells. Notably, B cell lymphoma subtypes with a dark zone signature are commonly more aggressive with a concomitant poor prognosis [52,53]. Hence, B cell lymphoma cells may benefit from acquired N-glycan motifs in their BCR by receiving sustained BCR activation via environmental mannose-binding lectins, as well as by the dark zone signature that is associated with this phenotype. Indeed, within the EZB subtype of DLBCL, patients with an acquired N-glycosylation motif in their CDR progressed faster than patients without an acquired N-glycosylation motif in this region [32].

Taken together, various B cell malignancies, especially FL, carry BCRs with acquired N-glycosylation motifs that are highly mannosylated. These glycan structures are ligated by mannose-binding lectins like DC-SIGN, that are expressed by cells in the tumor microenvironment, triggering continuous BCR signaling that aids in the progression and survival of the malignant B cells.

### 2.2. The Glycosphingolipid Globotriasosylceramide (Gb3/CD77) Is Highly Expressed on Burkitt’s Lymphoma Cells

Already in the 1980s it was recognized that a specific antigen expressed on Burkitt’s lymphoma cells (BL) could be detected using monoclonal antibody 38.13 [54,55]. This antibody was found to be specific for globotriasosylceramide, a glycosphingolipid also known as Gb3/CD77, that is composed of Gal(α1-4)Gal(β1-4)Glc(β1-1)Cer (Cer = ceramide). Gb3/CD77 expression on the surface of BL cells was found to be over 100 times higher than expression on other hematological malignancies [54,56]. Further in-depth analysis demonstrated that high expression of Gb3/CD77 is associated with high Gb3 synthetase activity [57], which could discriminate phenotypically different BL cell lines from each other. Gb3 contributes to the tumorigenic potential of BL cells, as a cell line clone that did express Gb3, but was unable to bind the Gb3 antibody (thus having a masked Gb3 antigen), demonstrated reduced tumorigenic potential when injected into mice [58]. Strong Gb3/CD77 expression has also been demonstrated in post-transplant lymphoproliferative disorder (PTLD), a post-transplantation condition in which B cells proliferate in an uncontrolled manner under the influence of immunosuppressants that may give rise to lymphoma [59].

Until recently it was not known how Gb3/CD77 regulates malignant B cell functioning and survival. However, Gb3/CD77 is also expressed on healthy GC B cells [60], and its function was recently studied in healthy murine GC B cells [60,61]. Modulation of Gb3/CD77, by knocking-out essential glycosyltransferases, did not impact the phosphorylation of CD79b and Syk [61]. Based on this data, it was concluded that Gb3/CD77 does not directly impact downstream BCR-signaling [61]. However, reduced phosphorylation of CD19, PI3K, and Akt, as observed in Gb3-deficient B cells, suggested that Gb3/CD77 signals via the CD19 pathway. Correspondingly, sorted Gb3-positive murine B cells demonstrated higher BCR-signaling as compared to sorted Gb3-negative cells. Mechanistically, Gb3/CD77 caused the dissociation of CD19 from chaperone CD81, allowing translocation of CD19 to the BCR complex. This stimulated the amplification of downstream CD19 signaling upon BCR stimulation (Figure 2B). Of note, Gb3/CD77 can also directly interact with CD19 [61], and has been described to impact the expression of CD19 [62] and CD20 [63]. Thus, Gb3/CD77 is a glycosylation pattern that is highly expressed on BL cells, where it likely contributes to BCR signaling. On the contrary, Gb3/CD77 is well-known for its ability to induce apoptosis once ligated [64]. In this respect, this glycolipid antigen is an interesting target for BL therapy (see Section 3).

### 2.3. Hypersialylation Negatively Impacts Survival of B Cell Lymphoma Patients

To determine glycosylation profiles of DLBCL samples, sections of formalin-fixed and paraffin-embedded biopsies (n = 57) were stained with various lectins, recognizing a broad range of N- and O-glycans [65]. Importantly, these sections were stained with and without prior neuraminidase treatment, a glycosidase that specifically cleaves off sialic acids from glycans. As expected, neuraminidase pre-treatment increased peanut agglutinin (PNA) binding, which is a commonly used positive control for neuraminidase-mediated release of sialic acids, as these sugar moieties block the PNA binding epitope (Galβ1-3GalNAc). Additionally, almost all samples reacted with Concanavalin A (ConA), a high mannose-N-glycan binding lectin, irrespective of neuraminidase treatment, i.e., 41 out of 47 (72%) untreated and 42 out of 47 (74%) treated samples. In contrast, no UEA-I and AAA epitopes were detected in any of the conditions, suggesting a lack of fucosylated blood group H O-glycans in these DLBCL samples. Lectins that bound much stronger to the neuraminidase-treated biopsies were Jacalin, MPA, HPA, ECA, and PHA-L, suggesting that core O-glycans, terminal GalNac and LacNac residues, and complex N-glycans are commonly sialylated in DLBCL. When categorizing the samples in a group that did not react with PHA-L at all (no epitope), a group that only reacted with PHA-L upon sialic acid removal (sialylated epitope), and a group where PHA-L binding did not change upon desialylation (non-sialylated epitope), it was demonstrated that overall survival chances were best for the group that expressed the non-sialylated epitope [65,66] (Figure 2C). Whether this impact on survival is specific for the PHA-L-binding epitope, or whether it is a more general sialylation effect is currently unknown. However, in BL, a reduced reactivity to PHA-L, PNA, and ConA was also associated with poor prognosis [67]. Since the survival curves (high versus low binder) of all three lectins looked exactly the same, it is likely that the association with survival is not epitope specific, but corresponds to a general glycosylation profile, most likely hypersialylation. In line with this data, hypersialylation was also increasingly detected in high metastatic lymphoma cells in comparison to less metastatic cells, in a mouse model [68]. In lymphoma, sialylation of the PHA-L-binding epitope was predominantly α2,6-coupled [66]. Also here, there was no correlation between *GntV* expression, the glycosyltransferase that mediates the formation of the PHA-L binding epitope, and PHA-L reactivity, again suggesting that the availability of this epitope depends on the extent of sialylation. Together, this data suggests that hypersialylation impacts the prognosis of patients with B cell lymphoma. Interestingly, increased levels of sialic acids were also detected in the serum of children with lymphoma during the onset of the disease as well as during relapse [69]. It is currently unknown where these sialic acids reside from. However, since sialic acid levels decreased during lymphoma therapy, it is tempting to speculate that the cancer cells themselves are the source of sialic acid. Further investigation of the impact of malignant B cell hypersialylation may be of interest for diagnostic, prognostic, and therapeutic purposes.

Of note, all the above staining experiments were performed on formalin-fixed and paraffin-embedded biopsies. Therefore, lectins may react with targets expressed both intracellularly and on the plasma membrane. As most immature, highly mannosylated N-glycans reside within the ER and Golgi [9], it is likely that the predominant ConA reactivity was intracellular. As cell–cell interactions rely on receptors and glycans expressed on the cell membrane, it is of high interest to analyze lectin-reactivity on non-fixed cells as well. Unfortunately, since lymphoma biopsies are commonly very small (needle biopsies), this may be challenging. However, advances in mass spectrometry-based glycomics, even on a single-cell level [70,71], and including plasma membrane labeling [72], may enable such analysis, also adding depth to the detected glycans, as lectins commonly recognize multiple glycan patterns [73].

## 3. Finding the Sweet Spot for the Treatment of B Cell Malignancies

Anti-cancer therapeutics commonly target proteins or pathways that are highly expressed by cancer cells. Therefore, the aberrantly or highly expressed glycan patterns on B cell malignancies (Section 2) may be interesting targets for therapy, as will be discussed in this section. This can be achieved using molecules that directly recognize the specific glycans, i.e., lectins, natural ligands, and antibodies, or modulating glycosylation patterns or glycosylation pathways using inhibitors and glycosidases. Furthermore, glycan-specific chimeric antigen receptor (CAR) T cell therapy is discussed, since this relatively new therapy has been a breakthrough for the treatment of refractory and relapsed B cell malignancies, and may be further developed by focusing on glycan targets.

### 3.1. Therapeutic Targeting of the BCR in B Cell Malignancies

#### 3.1.1. Targeting High-Mannose and Other Auto-Reactive BCRs

Inhibiting mannosylation on N-glycans would be a potential way to inhibit pro-survival signaling in B cell lymphoma cells with acquired high-mannose BCRs (see Section 2). Attempts have been made by using tunicamycin, which blocks N-glycosylation, which was moderately but significantly cytotoxic for various human B cell lymphoma cells [74,75]. Reducing N-glycosylation by knocking-out *OST-B,* a subunit that is crucial for N-glycosylation, also induced DLBCL cell death characterized by reduced BCR-glycosylation, BCR-clustering, and internalization [76]. However, since available mannosylation inhibitors lack specificity and are thus not suitable for clinical use, therapeutic targeting of highly mannosylated BCRs is currently challenging. Therefore, more efforts into the development of novel and more specific mannosylation inhibitors are warranted (see also Section 4: Perspectives).

**Figure 3:**
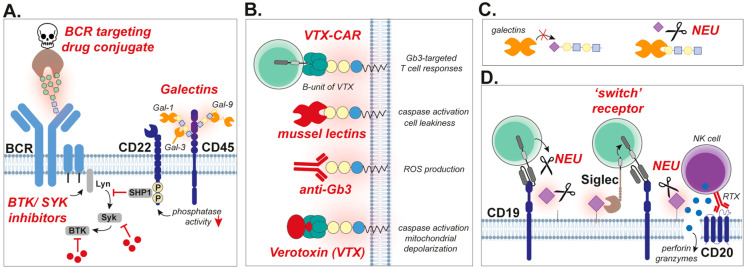
Sweet spots for the treatment of B cell malignancies. (**A**) Continuous BCR-signaling ensures survival in malignant B cells. The high-mannose BCR in malignant B cells can be targeted by drug conjugates, for instance, a DC-SIGN drug conjugate to induce cell death. BCR-signaling may also be reduced using galectins, which inhibit CD45 phosphatase activity, leading to enhanced phosphorylation of the inhibitory receptor CD22. In addition, important downstream proteins of the BCR (Syk, BTK) may be inhibited to reduce BCR-signaling. (**B**) Various options to exploit Gb3/CD77 expression on BL cells for therapy. Direct cytotoxicity can be induced by verotoxin (VTX, also known as ‘Shiga toxin’), resulting in caspase-dependent apoptosis. Cross-linking of Gb3/CD77 using (plate-bound) gb3-specific antibodies induces cell death that is dependent on the production of reactive oxygen species (ROS). Various types of mussel-derived lectins also specifically recognize Gb3/CD77, inducing caspase activation and cell leakiness. A chimeric antigen receptor (CAR) can be generated using the lectin-binding domain (B-unit) of VTX as a binding domain. (**C**) Galectins recognize beta-galactosides, which can be capped by sialic acids, preventing their interaction. Treatment with neuraminidase (NEU) cleaves off sialic acids, enabling galectin interactions, which may be used to modulate B cell adhesion. (**D**) Sialic acids are known for their inhibitory impact on immune responses. Removal of sialic acids with neuraminidase (NEU) can improve cancer cell lysis by natural killer (NK) cells upon treatment with rituximab (RTX, anti-CD20 antibody). Sialic acids can also be removed by cell-secreted NEU, for instance, in a NEU-producing chimeric antigen receptor (CAR) T cell format. In addition, sialic acids can be exploited by ‘switch receptors’, which convert inhibitory signaling as induced by sialic acids to activating signaling by coupling to a co-stimulator molecule.

However, BCRs from lymphoma cells may be specifically targeted using BCR autoantigens. In this respect, two studies focused on the identification of BCR autoantigens and used these peptides for targeted delivery of cytotoxic molecules. To this end, functional Ig heavy and light chains were amplified and used in a human protein microarray screen to determine binding partners, yielding SAMD14 and neurabin-1 as autoantigens in 8 out of 12 primary central nervous system lymphoma (PCNSL) samples (67%) [77]. By conjugating the BCR-binding epitope of neurabin-1 to truncated Pseudomonas exotoxin, a drug conjugate was produced that potently killed lymphoma cells with the neurabin-1-reactive BCR. A comparable approach in primary vitreoretinal lymphoma (PVRL) revealed SEL1L3 as an autoantigen in 3 out of 20 PVRL cases (15%), which also eradicated lymphoma cells once used in a drug conjugate [78]. Notably, SAMD14 and neurabin-1, as well as SEL1L3 auto-antigenic proteins, were hyper-N-glycosylated [77,78]. It would be of interest to target the highly mannosylated-BCR in FL with a similar ligand-based approach. Since lectins like DC-SIGN strongly bind to this aberrant BCR, DC-SIGN drug conjugates may be of particular interest (Figure 3A). To my knowledge, such DC-SIGN drug conjugates have not been designed to date, but a patent entitled ‘Dc-sign antibody drug conjugates’ has been published in 2020 by a pharmaceutical company [WO2020089811A1]. This patent describes the intended development of drug conjugates for the treatment of human diseases, among which is cancer; hence, DC-SIGN drug conjugates may be in development. In addition, BCR-signaling via the high-mannose/DC-SIGN interaction may be prevented by blocking mannoses or DC-SIGN. Mannoses can be blocked by anti-mannose antibodies [79], or mannose-based oligosaccharides that compete for DC-SIGN binding [80]. However, since mannoses are expressed everywhere throughout the body, this approach still needs a lot of fine-tuning before it will be suitable as an anti-lymphoma therapy. Of note, DC-SIGN targeting has especially been studied in inhibiting immune responses, using carbohydrates, antibodies, and quinolones [81,82]. As blocking DC-SIGN will also reduce dendritic cell-mediated immune responses, which is unfavorable in cancer, it is preferred to directly target the BCR and not its ligand DC-SIGN. In this respect, direct targeting of BCR-signaling using inhibitors of downstream kinases BTK and SYK is very effective in disrupting tumorigenic signaling in lymphoma cells [83,84] (Figure 3A). Although this approach does not directly target the aberrant glycan expression on the BCR, it has been proven to be effective in lymphoma cells with acquired N-mannosylated BCRs [39].

#### 3.1.2. Targeting BCR/CD45 with Galectins

CD45 is an important phosphatase expressed by all leukocytes, hence also known as ‘leukocyte common antigen’. B cells need CD45 to receive BCR-dependent survival signaling. Indeed, CD45 knockout B cells failed to induce downstream stimulatory signaling (e.g., PI3K, NfkB, and Erk) upon BCR-ligation [85]. Galectin-1 and galectin-3 have been demonstrated to interact with CD45 [86,87], which requires the presence of O-glycans and N-glycans for galectin-1 [87,88,89,90], and *C2Gnt-1*-dependent O-glycans for galectin-3 [86]. Similarly, galectin-9 was found to interact with CD45. In all cases, the interaction of the galectins with CD45 reduced its phosphatase activity, which resulted in the suppression of BCR-mediated signaling via the inhibitory molecule CD22 [86,88,91,92] (Figure 3A). As BCR-signaling is essential for the survival of B cell lymphoma cells [15,16], galectins may induce anti-lymphoma effects by inhibiting CD45 and/or BCR-signaling.

However, it should be noticed that the galectin-9 concentrations used to inhibit CD45/BCR-signaling are higher than needed to directly kill B cell lymphoma cells. Hence the reduction in CD45-mediated BCR-signaling may also be a result of measuring phosphatase activity in dying cells. Specifically, very recent work by our research group demonstrated that galectin-9 is cytotoxic for a broad range of B cell lymphoma cell lines, including, DLBCL, MCL, FL, and BL [93]. Sensitivity toward galectin-9 correlated with the basal protein expression levels of LC3B-I, a major player in the autophagy pathway, whereby cell lines with a higher basal level of autophagic flux were most sensitive toward this lectin. Galectin-9 inhibited the proper execution of autophagy, and was also able to eradicate chemoresistant B cell lymphoma cells. Also, both galectin-1 and galectin-3 are able to induce cell death in lymphoma cells, which, in contrast to galectin-9, relies on the apoptotic cell death pathway rather than autophagy [94,95]. Thus, galectins can induce cell death in malignant B cells via both apoptosis and autophagy pathways.

Notably, a difference between exogenously added (high) concentrations and endogenously present (low) concentrations of galectins may exist. Specifically, endogenous galectin-3 protected DLBCL cells against chemotherapy when bound to CD45, whereby the galectin-3 antagonist GCS-100 increased chemosensitivity [96]. Notably, a high degree of CD45 glycosylation has been associated with increased tumorigenicity [97]. Therefore, in-depth analysis of the specific glycan types of CD45 should be performed to understand how increased glycosylation results in increased tumorigenicity. Of note, CD45 exists in various isoforms, and it has been demonstrated that B cell lymphoma cells express a hyposialyated CD45-RA protein [98]. Furthermore, CML cells in blast crisis express high levels of *GnT-III* that increase the addition of bisecting GlcNac to CD45 [99]. Both hyposialylation and elevated GlcNacylation of CD45 may increase the interaction with galectins; hence, it is tempting to speculate that these specific B cell malignancies may be directly targeted using these lectins.

### 3.2. Targeting Gb3/CD77 to Treat Burkitt’s Lymphoma

#### 3.2.1. The Lectin ‘Verotoxin’ and Anti-Gb3/CD77 Antibodies

Verotoxin is a cytotoxin that is produced by certain *Escherichia Coli* and *Shigella Dysenteriae* strains, and hence also known as ‘Shiga-(like)toxin’. The B-unit of verotoxin comprises a lectin that specifically recognizes Gb3/CD77. As BL cells express high levels of Gb3/CD77, this cytotoxin has been used to induce BL-cell death [64] (Figure 3B). Mechanistically, verotoxin is transported to the ER, where it directly inhibits protein synthesis [100]. In addition, verotoxin activated caspase-8 and -3, leading to a reduction in mitochondrial membrane-potential and the release of cytochrome C. This activation of caspase-8 was dependent on the degradation of c-FLIP, an inhibitor of caspase-8 activation, leading to caspase-8-mediated activation of Bid, subsequent Bax activation, and finally cytochrome C release [101]. Furthermore, verotoxin treatment of BL cells activated Lyn and Syk kinases that are downstream of the BCR, and induced a patched BCR expression pattern [102], which corresponds to the finding that Gb3/CD77 is involved in BCR-signaling (see Section 2).

The activation of Gb3/CD77 using monoclonal antibodies also induced BL cell death [57] (Figure 3B). However, this required sufficient crosslinking, as Gb3/CD77-targeting antibodies (clone 38.28 or 1A4) were not cytotoxic in their soluble form, but only when immobilized on plates [57,102]. However, anti-Gb3/CD77 could increase the cytotoxic effect of a BCR-targeting antibody (anti-IgM) in soluble form [102]. Also, mechanistically, the induction of cell death by Gb3/CD77-targeting antibodies is different as compared to verotoxin. Specifically, anti-Gb3/CD77-induced cell death was caspase-independent and only resulted in partial depolarization of mitochondria. Furthermore, the cytotoxicity of anti-Gb3/CD77 was strongly reduced by ROS-scavengers, which had no impact on verotoxin-induced cell death.

As Gb3/CD77 is highly expressed on BL cells, it may also be targeted to specifically deliver other molecules. In this respect, an anti-CD3 antibody fragment (scFv OKT3), which induces T cell activation upon binding to T cell-expressed CD3, was coupled to the B-subunit of verotoxin [103]. This so-called ‘lectibody’ specifically and dose-dependently bound to both BL and T cells. Upon co-incubating BL cells with T cells, the lectibody induced specific killing of the BL cells, not affecting Gb3/CD77-negative cells or BL cells in which the generation of Gb3/CD77 was inhibited. T cells from co-incubations expressed more of the early and late T cell activation markers CD69 and CD25, respectively. Of note, in other Gb3-positive cancer types, verotoxin was also fused to a cytotoxic molecule to induce Gb3-specific cytotoxicity [104]. Thus, verotoxin on its own, or fusion molecules containing verotoxin or its Gb3/CD77-binding B-subunit on one arm and another immunostimulatory or cytotoxic molecule on the other arm, can be successfully used to target BL cells.

#### 3.2.2. Other Gb3/CD77-Specific Lectins

MytiLec is a lectin isolated from the mussel *Mytilus galloprovincialis*, and predominantly recognizes Gb3/CD77. Correspondingly, MytiLec induced dose-dependent cytotoxicity in Gb3/CD77-positive human BL cells Raji and Ramos, and not in the leukemic K562 cells that do not express Gb3/CD77 [105,106]. The increase in annexin-V binding, and thus phosphatidyl serine (PS) exposure, and PI incorporation suggest that MytiLec induced apoptosis and cell leakiness. Indeed, MytiLec activated caspase-3 and caspase-9 at 2 and 10 µg/mL, but not at higher concentrations (20 and 50 µg/mL) [106]. At these high concentrations, the treated B cell lymphoma cells were predominantly PI positive [105], suggesting that at lower concentrations MytiLec induces apoptosis, but at higher concentrations cells become disrupted, and likely die by necrosis/necroptosis. In addition, MytiLec induced TNFα secretion, which relied on mitogen-activated protein kinase (MAPK) pathways. In line with this, the MEK inhibitor U0126 inhibited the secretion of TNFα, although it did not prevent caspase activation. Similar results have been demonstrated using lectins isolated from the *Crenomytilus grayanus* mussel (CGL) and *Mytilus trossulus* mussel (MTL), which also recognize the Gb3/CD77 structure. Specifically, CGL-induced B cell lymphoma cell death was characterized by PS-exposure plus caspase activation and minimal PI incorporation, suggestive of caspase-dependent apoptosis [107]. Furthermore, both CGL and MTL induced cell cycle arrest, induced mitochondrial damage, and reduced colony formation [108]. Like MytiLec, CGL and MTL also activated the MAPK pathway. Another lectin isolated from catfish (*Silurus asotus*) eggs (SAL) that recognized Gb3/CD77, had no direct cytotoxic effect toward B cell lymphoma cells itself [109]. However, when B cell lymphoma cells were incubated with this lectin, their sensitivity toward chemotherapeutic drugs like vincristine and etoposide was significantly increased. Mechanistically, treatment with SAL reduced the expression of Multidrug Resistance 1 (MDR1) on both mRNA and protein levels. Taken together, Gb3/CD77 expressed on BL cells can be targeted by various Gb3-recognizing lectins to induce cell death, whereby most of them induce apoptosis (Figure 3B).

### 3.3. Other Glycosylation-Related Targets to Treat B Cell Malignancies

#### 3.3.1. Modulating Malignant B Cell Adhesion by Targeting Glycosylation

Adhesion is important for the survival of B cell malignancies (as reviewed by [110]). Since most adhesion proteins are highly glycosylated molecules, targeting glycosylation has the potential to impact B cell adhesion and survival. Indeed, overexpression of *FUT4* enhanced the expression of Le^x^ in acute lymphoblastic leukemia (ALL) cells (which can be either malignant T cells or B cells), which were, among others, carried by α5β1 integrins [111]. This glycan-modification increased the adhesion to fibronectin as well as the migration of ALL cells, as determined by a transwell assay. Further, pre-B leukemia and pre-B lymphoma cell lines highly expressed CD15s (sialyl Lewis x), a receptor that modulates cell adhesion by interacting with E-selectin [112]. CD15s expression was reduced by the O-glycan inhibitor benzyl-α-GalNac, whereas PDMP and swainsonine (glycosphingolipid and N-glycosylation inhibitor, respectively) had no effect, suggesting O-glycan-dependent CD15s expression. Indeed, CD15s expression correlated with *C2GnT* levels, a glycosyltransferase required for the generation of core-2 O-glycans. In line with this data, benzyl-α-GalNac impaired the adhesion of lymphoid cells to E-selectin positive cells, to a similar extent as anti-E-selectin and anti-CD15s blocking antibodies. Removal of N-glycans using swainsonine also increased the adhesion of lymphoma cells to galectin-1 [88,113], whereas inhibition of O-glycosylation using benzyl-α-GalNac enhanced adhesion to fibronectin [114]. Similar results were found for the adhesion of B cell lymphoma cells to galectin-8 and galectin-3 [114,115]. Of note, all these studies on galectin-mediated adhesion were performed by one and the same research group; hence, it is recommended to perform additional studies within other research groups to independently validate these findings. In addition, the clinical relevance of B cell lymphoma adhesion to galectin-1 is currently unclear. In this respect, galectin-1 was undetectable in 35 out of 42 NHL samples (83%) [116]. In contrast, galectin-1 is highly expressed in classical HL samples, where it associates with worse overall survival and event-free survival. Since galectin-1 was detected in both lymphoma-, immune-, and endothelial cells [116,117], its tumor-promoting effects may be a result of its immunomodulatory functions [118], or its impact on lymphoma-endothelial adhesion, the latter being required for lymphoma metastasis. Therefore, it is recommended to validate the impact of lymphoma glycosylation on adhesion to galectin-1 in a more clinically relevant setting using HL cells.

#### 3.3.2. Targeting N- and O-Glycosylation Pathways to Inhibit Malignant B Cell Survival

The expression of glycosyltransferase 1 domain-containing 1 (GLT1D1), an enzyme required for the transfer of glycosyl groups to proteins, was higher in B cell lymphoma cells as compared to normal samples, with *GLT1D1* expression being the highest in MCL [119]. In DLBCL, the expression of *GLT1D1* significantly correlated with progression-free survival and overall survival. This was attributed to the level of GLT1D1-dependent N-glycosylation of programmed dead ligand 1 (PD-L1), an important immunosuppressor. Specifically, *GLT1D1* expression positively correlated with the expression of glycosylated PD-L1 in both NHL cell lines and patient-derived DLBCL samples, whereby DLBCL patients with a poor prognosis expressed significantly higher levels of both GLT1D1 and PD-L1. The knockdown of GT1D1 downregulated PD-L1 expression and increased T cell activity (more IFN-γ and IL-2 secretion) and cytotoxicity. Of note, the importance of GLT1D1 could only be demonstrated in an in vivo mouse model using melanoma cells, as the CRISPR-mediated knockout of *GLT1D1* had a lethal phenotype in B cell lymphoma cells, again demonstrating its importance for B cell lymphoma survival. Also, in other cancer types it has been demonstrated that N-glycosylation of PD-L1 stabilizes its expression and suppresses anti-cancer T cell responses [120]. Therefore, it is of interest to develop efficient, specific, and safe N-glycosylation inhibitors for the treatment of B cell malignancies, and cancer in general.

O-GlcNAc transferase (OGT), the glycosyltransferase that adds GlcNAc to O-glycans, was found to be highly expressed in DLBCL cell lines and patient-derived tumor samples compared to healthy B cells [121]. Correspondingly, nuclear extracts of DLBCL samples contained elevated levels of GlcNAc compared to healthy B cells (O-GlcNAc is predominantly present intracellularly). Interestingly, high *OGT* expression levels significantly correlated with reduced progression-free survival of DLBCL patients. The inhibition of O-GlcNacylation by targeting *OGT* with siRNA reduced cell viability of DLBCL cell lines. Interestingly, similar results could be achieved by depleting the culture media of glucose, glutamine, and especially both, reflecting that the O-GlcNac-containing glycans are derived from these sugar sources. From a therapeutic point of view, this suggests that reducing O-GlcNAcylation may contribute to the successful treatment of DLBCL. Unfortunately, there are currently no clinically applicable OGT inhibitors available. However, the blood sugar levels of lymphoma patients may be regulated, e.g., by using insulin or metformin, to lower the levels of glucose and hence O-GlcNAc. In this respect it is interesting to mention that we observed that patients that receive intensive hematological treatments commonly experience episodes of hyperglycemia, i.e., too high blood sugar levels (>7.8 mM glucose) [122]. This may imply that blood glucose levels should be tightly regulated in patients with B cell lymphoma during treatment to prevent increased O-GlcNAcylation, something that is currently under investigation within our department. Together, it is evident that the inhibition of certain glycosylation pathways negatively impacts the survival of B cell malignancies in vitro. However, for clinical translation, it is warranted that more specific and non-toxic glycosylation manipulators are developed.

#### 3.3.3. Glycan-Focused Chimeric Antigen Receptor (CAR) T Cell Therapy

A recent breakthrough in the treatment of B cell malignancies is chimeric antigen receptor (CAR) T cell therapy, which increased the 5-year survival time of refractory and relapsed large B cell lymphoma patients from ~10% to ~50% [123]. To obtain CAR T cells, the patient’s own T cells are harvested from the blood and genetically engineered to express a receptor that recognizes a target on the cancer cells, being CD19 for B cell malignancies [124]. After expansion, the CAR T cells are given back to the patient, after which cancer cells are being eliminated. The CD19-CAR is using an ScFv antibody-fragment to recognize the CD19 protein. Interestingly, several attempts have been made to make glycan-specific CAR T cells, using both ScFv and lectins as activating binding domains. For the targeting of BL, three different lectin-based CARs targeting Gb3/CD77 were developed [125]. Specifically, *Mytilus galloprovincialis* lectin (see above other Gb3/CD77 lectins part), the B-subunit of Shiga toxin (see above verotoxin part), and LecA isolated from *Pseudomona aeruginosa* were used as antigen-binding domains in these CARs. From all these three constructs, the Siga-CAR (or verotoxin-CAR; VTX-CAR, Figure 3B) was clearly cytotoxic for Gb3-positive but not Gb-negative B cell lymphoma cells, although the benchmark CD19-CAR was more effective. The two other lectin-CARs hardly demonstrated any cytotoxic activity, which, in the case of the *Mytilus-*CAR, may be due to very low expression levels of the CAR on the T cells. In contrast, the LecA-CAR was the highest expressed among all CARs, even higher than the CD19-CAR. Upon sorting the T cells that were positive for the CAR, and performing cytotoxicity assays with ‘pure’ CAR T cells, all CARs induced specific killing. Here, the CD19-CAR was most effective, followed by the VTX-CAR, LecA-CAR, and *Mytilus-*CAR. Although the CD19-CAR seems to outperform the lectin CARs in terms of in vitro cytotoxicity, it is unknown how these CARs behave in vivo and in patients. Especially, since high activity and also affinity may also result in faster T cell differentiation and exhaustion, reducing the CAR T cell persistence [126]. In this respect, when targeting CD7-positive leukemia, the ligand-based (K12) CAR T cell outperformed the scFv-based (scFvCD7) CAR T cell in a sequential killing assay, as demonstrated by our research group [127]. Therefore, it is recommended to directly compare scFv-CARs to lectin-based CARs in sequential assays and in vivo experiments.

Also, in other cancer types, lectin-based CARs have been designed, among which are recombinant C-type lectin (CD301) CARs in osteosarcoma [128] and banana lectin (H84T) to target high mannose in pancreatic cancer [129]. In addition, ScFv-based CARs using antibody fragments that recognize glycan-structures were used, for example, anti-stage-specific embryonic antigen-4 CARs in pancreas cancer [130], anti-GalNac (also known as Tn antigen) CARs [131], and anti-sialylated O-glycan sialyl-Thomsen Nouveau antigen (STn) CARs in multiple cancer types [132]. Since all these CARs were able to induce cancer cell death, it may be of interest to design CARs toward aberrantly expressed glycans on B cell malignancies (see Section 2), for instance, DC-SIGN CARs and the above-mentioned VTX-CARs. However, it should be noticed that specificity may be an issue with such CARs, since even though certain glycans may be highly expressed on cancer cells, they are commonly not absent on other (healthy) cells, and hence the glycan-CARs may induce on-target off-tumor cytotoxicity.

#### 3.3.4. Improving Therapy by Targeting Sialic Acids

As evidenced from Section 2, a high level of sialic acids seems to correlate with worse overall survival in B cell malignancies. Sialic acids are known for their negative impact on immune responses (as reviewed by [133]). Therefore, the removal of sialic acids may improve cancer therapy, especially immunotherapy. Furthermore, sialic acids are located at the external end of the glycan tree; hence, the removal of sialic acids will also uncover other glycan structures that are capped by the sialic acids.

As described above, lymphoma cell adhesion seems to be (at least partially) regulated by galectins. Galectin-binding epitopes, like poly-LacNac, are also commonly sialylated. Indeed, the removal of sialic acids using neuraminidase increased the binding of DLBCL, BL, and ALCL cells to galectin-1, strengthening adhesion [88,113] (Figure 3C). Correspondingly, the KO of *ST6Gal1* enhanced adhesion to galectin-1, whereas this was inhibited by resialylation using recombinant ST6Gal1 and CMP-Neu5Ac [88]. Therefore, modulating sialylation of lymphoma cells may impact their ability to adhere, and hence may influence metastatic potential.

Another aspect in the context of malignant B cell therapy in which sialic acids have been implicated is in the efficacy of rituximab (RTX), an anti-CD20 monoclonal antibody that is used in first-line treatment of most B cell malignancies (R-CHOP; rituximab plus cyclophosphamide, (hydroxy)doxorubicin, vincristine, and prednisone). Here, rituximab contributes to the induction of immune responses via antibody-dependent cell-mediated cytotoxicity (ADCC) and antibody-dependent cell-mediated phagocytosis (ADCP). Patient-derived CLL samples that were resistant to rituximab-induced CDC expressed higher levels of terminal α2-6-linked sialic acids [134]. Correspondingly, the removal of sialic acids using neuraminidase increased CDC upon rituximab treatment (Figure 3D). Of note, also, the glycosylation of rituximab itself impacts its efficacy, whereby defucosylation increased its efficacy [135].

Sialic acids are known inhibitors of T cell responses [136], and therefore also impair CAR T cell responses. To circumvent this problem, a neuraminidase-secreting CD19-CAR T cell was developed that reduced sialic acid levels (Figure 3D), improving overall activity both in vitro and in vivo [137]. This approach may also guarantee local production and secretion of neuraminidase, improving its efficacy, as sialic acids can be found readily throughout the bloodstream. Another interesting approach in the context of T cell responses is the use of ‘chimeric switch receptors’ (CSR), which refers to receptors that normally have inhibitory effects, but are ‘switched’ to activators by connecting them to co-stimulatory domains. To target sialic acids, siglec-9, a sialic acid-binding inhibitory receptor, was connected to the co-stimulatory molecule CD28 or 4-1BB [138] (Figure 3D). This CSR increased T cell activation and demonstrated in vivo antitumor activity towards various solid tumors. Hence, adding such sialic acid-targeting CSRs to CD19-CARs may increase their efficacy towards B cell malignancies. Furthermore, such CSRs may also be generated towards other glycans expressed on malignant B cells, like Gb3/CD77.

Thus, the removal of sialic acids is a promising way to increase therapeutic efficacy towards B cell malignancies, which can be achieved by enzymatic removal using neuraminidase or targeting the sialylation machinery. However, it may be challenging to achieve high enough concentrations of neuraminidase at the site of the tumor, and (like N- and O-glycan inhibitors) clinically suitable inhibitors of the sialylation pathway are not yet available, and hence should be developed.

## 4. Perspectives: Clinical Translation of Glycan-Targeting in B Cell Malignancies

As is evident from this review, glycosylation is a target with potential for the treatment of malignant B cells. However, several hurdles still have to be taken before clinical implementation can be realized. The standard treatment option for B cell malignancies remains chemotherapy; hence, novel glycan-targeting approaches may be combined with this treatment regimen to improve therapeutic responses. A first strategy that has the potential to reach the clinic is the glycan-mediated targeting of CD22, a Siglec family member (Siglec-2) that is expressed on B cells. For example, doxorubicin was targeted to lymphoma cells using nanoparticles that were coated with high-affinity glycan ligands for CD22 [139,140]. This resulted in lymphoma cell death both in vitro and in vivo, and also eliminated patient-derived B cell leukemia and lymphoma cells ex vivo. To date, no clinical trials for the safety and efficacy of glycan-targeted CD22 have been initialized. However, CD22-targeting antibodies like Epratuzumab have been tested in clinical trials [NCT0030182, NCT01262365, NCT01261793], which proved to be safe and well-tolerated in lymphoma as well as lupus patients. Furthermore, by using a high-affinity glycan ligand for CD22, malignant B cells could be targeted without harming healthy B cells, as demonstrated for NK-cell-based immunotherapy [141]. Hence, CD22-targeted delivery of chemotherapy using cancer-specific high-affinity glycans may have clinical potential. This is in contrast to currently available glycan-based targeting of high-mannose N-glycans using DC-SIGN as well as Gb3/CD77 using lectins like Shiga toxin, as too high toxicities are expected. Indeed, Shiga toxin is well known for its severe gastrointestinal effects, which may be life-threatening [142]; hence, systemic application will lead to severe side-effects. As DC-SIGN is also a ligand for ICAM-3 expressed on T cells [143,144], systemic use of DC-SIGN potentially also impacts T cell responses. Thus, before these types of glycan targeting can be translated to the clinic, more specific modalities have to be developed. Also, the systemic application of lectins is expected to lead to side effects, as the glycans that they recognize, although elevated on malignant B cells, are also present on healthy cells. For instance, galectin-9 was cytotoxic for both malignant B cells as well as healthy B cells [93]. However, galectin-9 may be administered at certain moments during treatment in which the immune system has to be suppressed and/or eradicated, like during lymphodepletion prior to hematopoietic stem cell transplantation or CAR T cell therapy.

Interestingly, the activity of the clinically used polatuzumab vedotin (Polivy), an antibody-drug conjugate directed to the BCR subunit CD79B [145], was demonstrated to be dependent on glycosylation [146]. Specifically, sialic acids coupled to N-glycans were found to block the epitope of Polivy. Sialic acids are well known for their ability to block immune responses [147], and were reported to inhibit the efficacy of rituximab by reducing the activation of the complement pathway in CLL patients [134]. Both these examples argue for a combinational treatment with sialic acid-reducing agents. However, commonly used sialic acid inhibitors are not suitable for clinical use. Indeed, systemic blockade of sialylation using the sialic acid mimetic 2,4,7,8,9-pentaacetyl-3Fax-Neu5Ac-CO2Me (Ac_5_3F_ax_Neu5Ac) induced strong liver and kidney dysfunction in mice [148]. To circumvent systemic side-effects, Ac_5_3F_ax_Neu5Ac was given intratumorally in another study, which effectively reduced sialylation in melanoma-bearing mice without clear signs of cytotoxicity [149]. However, for most clinical treatments in humans, it is not possible to perform intratumor injections, especially not in B cell leukemias and lymphomas. Local administration of sialic acid inhibitors may be achieved by the use of nanocarriers that are coated with tumor-targeting antibodies. This has proven to be successful and safe in melanoma-bearing mice, where Ac_5_3F_ax_Neu5Ac was encapsulated into poly(lactic-co-glycolic acid) nanoparticles coated with tumor-targeting antibodies [150]. To translate this to malignant B cells, tumor targeting may be achieved using rituximab-guided (anti-CD20) nanoparticles, for which we have provided proof-of-concept recently [151]. A second interesting approach may be found in agents that are inactive systemically, but can be activated once at the tumor site. This was reported for SiAFNEtoc, another sialic acid inhibitor, that was conjugated to a photolabile protecting group, yielding a UV-activatable agent [152]. UV-SiAFNEtoc demonstrated proof-of-concept for in vitro cell cultures. Next, suitability testing for in vivo use would be of great interest, especially since B cell lymphomas are commonly irradiated, and hence radiation-activated prodrugs [153] may also be of interest.

Notably, glycan-modification may also be particularly suitable for CAR T cell therapy, since these cells are produced outside the body, and hence systemic side-effects of inhibitors can be circumvented. Importantly, for such inhibitors to work in the patient, their impact should be long-lasting and remain even after withdrawal of the inhibitor. SiAFNEtoc may be a candidate for sialic acid inhibition, since a 72h pre-treatment of prostate cancer cells with this agent could significantly reduce their ability to form tumors for 15 days up to 6 weeks in two different in vivo models [154]. For CAR T cell therapy, it is known that early responses, measured with FDG-PET imaging at 30 days after CAR T cell infusion, associate with overall CAR T cell responses in ~70% of the cases [155]. Further, peak expansion of CAR T cells is found around day 10 post-infusion [156]. Hence, the CAR T response that dictates eventual outcome takes place at early time-points, which may fall within the time range that SiAFNEtoc pre-treatment can retain reduced sialic acid levels. In addition, the glycome of CAR T cells may be permanently modified by the knockout (or overexpression) of glycosylation-related genes, which has been proven successful in several preclinical studies [157,158,159]. Among these, CAR T cells with a deletion of *SPPL3*, inducing a more extensive N-glycosylation phenotype, already entered a phase I clinical trial, and reached the safety primary endpoint and complete remission in six out of nine subjects with B cell malignancies [[159], NCT06014073]. Notably, although CRISPR-cas9 mediated knockout is currently the predominant technique of choice, epigenetic editing may be a safer option for clinical application, for which proof-of-concept was recently demonstrated in CAR T cells [160,161,162]. When comparing glycan-targeting CARs to glycan-modified CARs, glycan-targeting CARs may face more on-target off-tumor toxicities due to the widespread presence of glycans throughout the human body. To avoid this, cancer-specific glycans should be targeted, like the sialyl-Tn-targeting CAR T cell that was recently published for solid tumors [163]. In order to find such glycans that are exclusively expressed on malignant B cells, more in-depth characterization of glycan expression profiles is warranted, which may take a great leap forward in the coming years with the development of single-cell glycomics and spatial glycomics [70,71,164,165], the latter being of especial interest for lymphoma biopsies.

## 5. Conclusions

Malignant B cells express various glycosylation patterns that discriminate them from healthy B cells and other cells. These aberrant glycans are therefore of interest for diagnostics, prognostics, and therapy. Therapeutic targeting can be achieved by (cytotoxic) lectins, glycosylation inhibitors/modulators, or glycan-targeting immunotherapeutics, like CAR T cells. However, for clinical translation, specificity and toxicity are currently the biggest issues to be solved. Therefore, more in-depth characterization of glycan expression profiles is warranted, a field that is expected to advance in the coming years, and may yield novel cancer-specific targets. Together, it is anticipated that glycosylation will become a more frequently used target for therapeutics and diagnostics for B cell malignancies in the (near) future.

## Figures and Tables

**Table 1:** Acquired N-glycan motifs in B cell malignancies. (**A**) Acquired N-glycan motifs in the heavy chain of the BCR-Ig of FL samples. (**B**) Acquired N-glycan motifs in the light chain of the BCR-Ig of FL samples. (**C**) Acquired N-glycan motifs in the BCR-Ig of other (malignant) B cells. **Frequency**: Number of samples that have at least one acquired N-glycosylation motif. **Isotype:** Isotype of Ig in which the acquired N-glycosylation motif was detected, with the most common isotype in bold. **Location:** Location in the Ig in which the acquired N-glycosylation motif was found, with the most frequent location in bold. **$** FL and FLIS. * PCFCL.

**(A)** **Acquired N-glycan motifs in the heavy chain of the BCR-Ig of FL samples**			
**Reference**	**Frequency Acquired** **Frequency Acquired N-Glycan Motif**	**Isotype of BCR-Ig**	**Location Acquired** **N-Glycan Motif**
Zhu et al., 2002 [23]	16 out of 17 (**94%**) 55 out of 70 (**79%**)	Ig	CDR1, **CDR2**, CDR3, FR1, FR3
Belessi et al., 2002 [24]	4 out of 10 (**40%**)	Ig	**CDR2,** FR1, FR2
Zabalegui et al., 2004 [19]	24 out of 24 (**100%**)	IgM, **IgG**	CDR2, **CDR3**, FR3
Radcliffe et al., 2007 [25]	6 out of 6 (**100%**)	IgM, **IgG**	CDR2, **CDR3**, FR1
McCann et al., 2008 [26]	7 out of 7 (**100%)**	Ig	CDR1, **CDR2**, CDR3, FR1, FR3
Mamessier et al., 2015 [27] **$**	1 out of 1 (**100%**)	Ig	
Koning et al., 2019 [28] *	12 out of 18 (**67%**)	IgA, IgM, **IgG**	CDR1, **CDR2**, **FR3**
Odabashian et al., 2020 [21]	6 out of 6 (**100%**)	Ig	CDR1, CDR2, **CDR3**, FR2, GFR3
Lim et al., 2021 [29]	1 out of 1 (**100%**)	IgM	CDR1, **CDR3**
Haebe et al., 2023 [20]	15 of the 17 (**88%**)	**IgM**, IgG	CDR1, CDR2, **CDR3**, FR2, FR3
**(B) Acquired N-glycan motifs in the light chain of the BCR-Ig of FL samples**			
**Reference**	**Frequency Acquired** **N-Glycan Motif**	**Isotype of BCR-Ig**	**Location Acquired** **N-Glycan Motif**
Zhu et al., 2002 [23]	10 out of 17 (**59%**)	Ig	
Belessi et al., 2002 [24]	0 out of 10 (**0%**)	Ig	
Radcliffe et al., 2007 [25]	2 out of 6 (**33%**)	Ig	CDR2, FR4
McCann et al., 2008 [26]	2 out of 7 (**29%**)	Ig	CDR1, CDR3
Koning et al., 2019 [28] *	12 out of 18 (**67%**)	IgA, IgM, **IgG**	**CDR1**, CDR2, CDR3, FR1, **FR3**
Lim et al., 2021 [29]	0 out of 1 (**0%**)	IgM	
Haebe et al., 2023 [20]	7 out of 17 (**41%**)	**IgM**/IgG	
**(C) Acquired N-glycan motifs in the BCR-Ig of other (malignant) B cells**			
**Reference**	**Subtype**	**Frequency Acquired N-Glycan Motif**	
**Normal B cells**			
Zhu et al., 2002 [23]	-	7 out of 75 (**9%**)	
**Burkitt’s lymphoma**			
Zabalegui et al., 2004 [19]	BL	1 out of 4 (**25%**)	
Zhu et al., 2003 [30]	endemic BL	14 out of 17 (**82%**)	
Zhu et al., 2003 [30]	sporadic BL	10 out of 23 (**43%**)	
Zhu et al., 2003 [30]	AIDS-BL	3 out of 9 (**33%**)	
Forconi et al., 2004 [31]	AIDS-BL	5 out of 12 (**42%**)	
Zhu et al., 2003 [30]	Iranian-BL	4 out of 4 (**80%**)	
**Diffuse large B cell lymphoma**			
Zhu et al., 2002 [23]	DLBCL	13 out of 32 (**41%**)	
Zabalegui et al., 2004 [19]	DLBCL	0 out of 6 (**0%**)	
Koning et al., 2019 [28]	DLBCL	0 out of 8 (**0%**)	
Chiodin et al., 2021 [32]	DLBCL	GCB: 55 out of 92 (**60%**), ABC: 23 out of 180 (**13%**)	
Forconi et al., 2004 [31]	AIDS-DLBCL	11 out of 24 (**46%**)	
Forconi et al., 2004 [31]	PT-DLBCL	3 out of 15 (**20%**)	
Koning et al., 2019 [28]	PC-DLBCL	0 out of 8 (**0%**)	
**Other lymphoma**			
Zhu et al., 2002 [23]	CLL	5 out of 40 (**13%**)	
Zabalegui et al., 2004 [19]	MCL	1 out of 3 (**30%**)	
Zabalegui et al., 2004 [19]	SLL	0 out of 4 (**0%**)	
Zhu et al., 2003 [30]	MALT	3 out of 34 (**9%**)	
Forconi et al., 2004 [31]	AIDS-NHL	16 out of 36 (**44%**)	
Forconi et al., 2004 [31]	PTLD	4 out of 19 (**21%**)	
Forconi et al., 2004 [31]	P-PTLD	1 out of 4 (**25%**)	
**Multiple myeloma**			
Zhu et al., 2002 [23]	MM	5 out of 64 (**8%**)	
Belessi et al., 2002 [24]	MM	2 out of 17 (**12%**)	

## Data Availability

Not applicable.

## Abbreviations

The following abbreviations are used in this manuscript:

ABC	Activated B Cell-like
Ac_5_3F_ax_Neu5Ac	2,4,7,8,9-pentaacetyl-3Fax-Neu5Ac-CO2Me
ADCC	Antibody-Dependent Cell-Mediated Cytotoxicity
ADCP	Antibody-Dependent Cell-Mediated Phagocytosis
AIDS	Acquired Immunodeficiency Syndrome
ALL	Acute Lymphoblastic Leukemia
Asn	Asparagine
BCR	B cell Receptor
BL	Burkitt’s Lymphoma
CAR	Chimeric Antigen Receptor
CDR	Complementarity-Determining Region
CLL	Chronic Lymphocytic Leukemia
ConA	Concanavalin A
CSR	Chimeric Switch Receptor
DC-SIGN	Dendritic Cell-Specific Intercellular Adhesion Molecule-3-Grabbing Non-integrin
DLBCL	Diffuse Large B Cell Lymphoma
FL	Follicular Lymphoma
GalNAc	N-acetylgalactosamine
GC	Germinal Center
GCB	Germinal Center B Cell-Like
GlcNAc	N-acetylglucosamine
HL	Hodgkin’s Lymphoma
Ig	Immunoglobulin
MBL	Mannose-Binding Lectin
MCL	Mantle Cell Lymphoma
moDC	Monocyte-Derived Dendritic Cells
MR	Mannose Receptor
NEU	Neuraminidase
NHL	Non-Hodgkin Lymphoma
NK	Natural Killer
OGT	O-GlcNAc Transferase
PCFCL	Primary Cutaneous Follicle Center Lymphoma
PCNSL	Primary Central Nervous System Lymphoma
PD-L1	Programmed Dead Ligand 1
PNA	Peanut Agglutinin
PS	Phosphatidyl Serine
PTLD	Post-Transplant Lymphoproliferative Disorders
PVRL	Primary Vitreoretinal Lymphoma
RTX	Rituximab
Ser	Serine
SLL	Small Lymphocytic Lymphoma
Thr	Threonine
